# Spatial and seasonal foraging patterns drive diet differences among north Pacific resident killer whale populations

**DOI:** 10.1098/rsos.240445

**Published:** 2024-09-18

**Authors:** Amy M. Van Cise, M. Bradley Hanson, Candice Emmons, Dan Olsen, Craig O. Matkin, Abigail H. Wells, Kim M. Parsons

**Affiliations:** ^1^ North Gulf Oceanic Society, Visiting Scientist at Northwest Fisheries Science Center, National Oceanic and Atmospheric Administration, Seattle, WA, USA; ^2^ School of Aquatic and Fishery Sciences, University of Washington, Seattle, WA, USA; ^3^ Northwest Fisheries Science Center, National Marine Fisheries Service, National Oceanic and Atmospheric Administration, Seattle, WA, USA; ^4^ North Gulf Oceanic Society, Homer, AK, USA; ^5^ Lynker Technologies, Leesburg, VA, under contract to Northwest Fisheries Science Center, National Marine Fisheries Service, National Oceanic and Atmospheric Administration, Seattle, WA, USA

**Keywords:** diet, foraging, killer whale, metabarcoding, faecal, top marine predator, endangered

## Abstract

Highly social top marine predators, including many cetaceans, exhibit culturally learned ecological behaviours such as diet preference and foraging strategy that can affect their resilience to competition or anthropogenic impacts. When these species are also endangered, conservation efforts require management strategies based on a comprehensive understanding of the variability in these behaviours. In the northeast Pacific Ocean, three partially sympatric populations of resident killer whales occupy coastal ecosystems from California to Alaska. One population (southern resident killer whales) is endangered, while another (southern Alaska resident killer whales) has exhibited positive abundance trends for the last several decades. Using 185 faecal samples collected from both populations between 2011 and 2021, we compare variability in diet preference to provide insight into differences in foraging patterns that may be linked with the relative success and decline of these populations. We find broad similarities in the diet of the two populations, with differences arising from spatiotemporal and social variability in resource use patterns, especially in the timing of shifts between target prey species. The results described here highlight the importance of comprehensive longitudinal monitoring of foraging ecology to inform management strategies for endangered, highly social top marine predators.

## 1. Introduction

In highly social and long-lived top marine predators, including many cetacean species, important ecological strategies such as diet preferences, foraging locations, and foraging techniques are culturally learned [1–5]. Cultural learning can increase the resilience of a species or population on evolutionary timescales [1]; conversely, cultural rigidity can decrease resilience to rapid changes to the environment [6,7]. Rapid changes in the prey resource landscape, on the scale of multiple decades, could challenge the resilience of a social group that relies heavily on the knowledge of specific cultural leaders to respond to novel ecosystem shocks [7] by balancing ecological trade-offs between resource availability and the cost of resource acquisition [8–11].

When top marine predator populations are endangered, managers are faced with a diversity of challenges to understanding and managing the drivers of population declines. Data limitations and limited knowledge of synergistic interactions among threats often confound efforts to quantify the effects of multiple stressors. Our understanding of the effect of social structure and cultural learning on a population’s resilience to environmental change is often confounded by the research challenges of small sample size, lagged demographic responses, and long generation times [12–14]. The wide geographic ranges of many top marine predators often limit the collection of data and biological samples, especially when paired with limited availability of funding to support longitudinal research and monitoring of top marine predators, which further impede efforts to quantify the effects of stressors such as prey limitation and habitat degradation [15–17]. Field studies of ecological behaviours in most top marine predators are often inhibited by remote, inaccessible habitats and elusive behaviours, which makes it difficult or impossible to directly observe, e.g. foraging behaviour. Because of these factors, it is often true that the proximate causes of demographic shifts and population decline are not well understood, leaving policymakers struggling to identify and implement management strategies that will promote the recovery of endangered populations.

Killer whales (*Orcinus orca*) are known for their highly specialized foraging strategies and diet preferences [18]. In the northeastern Pacific Ocean there are three partially sympatric populations of the resident killer whale (RKW) ecotype: southern Alaska resident killer whales (SARKW, central–southeast Alaska), northern resident killer whales (NRKW, southeast Alaska–coastal WA) and southern resident killer whales (SRKW, northern BC–central CA). RKW are known to prey on fish, specifically salmonids (*Oncorhynchus* spp.), and are thought to rely heavily on Chinook salmon (*Oncorhynchus tshawytscha*, hereafter ‘Chinook’), chum (*Oncorhynchus keta*) and coho salmon (*Oncorhynchus kisutch*). Although all three of these populations have overlapping ranges, social and cultural practices affecting mating decisions are hypothesized to have caused genetic isolation and limited gene flow among them [19].

Since population monitoring began with the advent of photo-ID in the early 1970s (e.g. [20–23]), the demographic trajectory of the SRKW has been markedly different from those of the NRKW and SARKW [20]. The SRKW population contracted by roughly 30% in the late 1960s due to several years of live capture for the aquarium industry, and by 1971 was reduced to 67 individuals [24]. Since then, their abundance has not recovered, and today hovers around 70–75 individuals [25]. SRKW were listed as endangered under the US Endangered Species Act (ESA) in 2005 and by the Committee on the Status of Endangered Wildlife in Canada (COSEWIC) and the Species at Risk Act (SARA) in Canada in 2003 [26–28]. The population faces multiple threats, including limited prey resources, exposure to persistent organic pollutants (POPs), inbreeding effects, vessel traffic and noise pollution. In contrast, the SARKW and NRKW populations are currently considered to be robust, both exhibiting positive trends in population size [29,30]. The relative stability of the NRKW and SARKW populations may be due in part to relatively stable prey resources [31]. SARKW specifically are known to exhibit regional and temporal variability in the proportion and preferred species that supplement the diet [31,32], and salmon populations in central–southeast Alaska are not expected to experience significant declines related to climate change [33], which is likely to contribute to future stability of the SARKW population.

Several studies examining the effects of salmon abundance on SRKW demographics suggest that prey limitation, specifically Chinook availability, is a threat to SRKW survival, along with other known threats such as disturbance [34,35] and inbreeding depression [36]. One study focused on the SRKW J pod found that trends in SRKW body condition are indicative of individual survival likelihood and have been associated with prey availability [37]. Between 1979 and 2003, mortality rates in SRKW were correlated with the coast-wide abundance of Chinook salmon [38,39]; e.g. decreased Chinook abundance correlated with increased SRKW mortality. Fecundity in SRKW is lower than in NRKW across all age groups, and annual fecundity rates have been correlated with annual abundance of Chinook salmon [40]; scientists have documented increased birth rates in SRKW in years following a year of high Chinook abundance [38]. Declining birth rates can be attributed to a lack of prey resources or increasing prey heterogeneity in many wild populations [41–45].

Research on the diet of SRKW conducted since the late 1990s supports the hypothesis that Chinook salmon are the primary prey source of this population during the summer months (May–September) when these animals are in the Salish Sea and when the majority of data have been collected [18,46–49]; chum salmon also make up a significant portion of the diet in the later summer months. Data from prey tissue samples collected at predation events, primarily during summer months, indicate that Chinook may make up 70% or more of the SRKW diet, or as much as 400 000 Chinook annually [18,48,50,51]. Data from faecal samples indicate Chinook may be an even larger portion of the diet at some times of the year, but also indicate greater diet diversity than detected via prey tissue samples [47,49]. Chinook in the Salish Sea are currently considered to be at 30–50% of their historical abundance, including both wild and hatchery-born fish [52,53], and are decreasing in size due to a variety of factors that including fishing and habitat degradation, as well as the effect of predation by SRKW [54–57]. Further, Chinook abundance may continue to decline in abundance in this region due to the effects of climate change [33,58,59].

Despite focused research spanning approximately two decades, our current understanding of the diet of SRKW (and resident killer whales more broadly) is based on samples collected primarily during the summer and early autumn in the Salish Sea, with a small but growing number of samples representing other seasonal and geographic strata [47,49]. These studies indicate that the SRKW diet may include a greater variety of prey species than previously understood, including significant diet contributions from chum and coho salmon, as well as non-salmonid species such as Pacific halibut (*Hippoglossus stenolepis*) and lingcod (*Ophiodon elongatus*) [47,49]. These findings highlight the need to examine seasonal fluctuations in diet during non-summer months, especially as they relate to survival and fecundity. To date, studies of SRKW diet have largely been limited to population-level inference and have not examined the probable effect of pod-specific foraging strategies on temporal diet variability. Pod-specific foraging strategies in response to environmental variability are a key factor linking prey availability to pod-specific likelihood of survival and reproduction [37,40], therefore describing the fine-scale differences in foraging behaviour among SRKW pods is an important step to improving SRKW management. Further, the previous correlation between Chinook abundance and SRKW population dynamics has decoupled since 2010–2012 [60], suggesting possible shifts in SRKW foraging behaviour in response to prey limitation, or the possibility that previous studies did not capture the full repertoire of SRKW foraging ecology.

In this study, we build on previously published datasets and analyses of SRKW diet [47,49] to compare variability in the seasonal diet and foraging habits of SARKW and SRKW using faecal samples collected between 2011 and 2021 and provide new insights into factors contributing to the contrasting trends in abundance of these two populations. Further, we examine high-resolution foraging patterns in SRKW, including pod-specific and seasonal variability in diet preferences, to inform the management of target prey resources.

## 2. Methods

### 2.1. Sample collection

Faecal samples were collected during multi-objective field projects focused on SRKW or SARKW, either opportunistically or using targeted focal follows of groups or individuals. Faecal samples were detected visually, by odour, or by the presence of avian scavengers. Samples were collected from the water surface using a fine mesh net with a long handle and were either scooped directly into a sterile vial (polypropylene or glass) or, if the sample was small, wiped from the net with sterile gauze and placed into a sterile vial. Samples were stored in a cooler on ice packs for up to 8 h until being transferred to a −20°C or −80°C freezer. Sample metadata, including collection date and sampling location, are available in electronic supplementary material, table S1; approximate sampling locations and annual sampling effort are shown in electronic supplementary material, figure S1.

Samples from SRKW were collected under NOAA permits 781-1824, 16163 and 21348, and collection protocols were reviewed and approved under IACUC protocols NWAK-18-01 and A/NW 2014-02 (NWFSC ESA/MMPA 5 year Marine Mammal Research Permit). SARKW faecal samples were collected under NMFS permits 15616 and 20341.

Samples collected from the same individual on the same date were considered to be biological replicates. Similarly, some samples were subdivided and sequenced multiple times to create a set of technical replicates in order to examine potential variability introduced during sample processing and library preparation.

### 2.2. Prey metabarcoding

Extraction and amplification protocols for new faecal samples were consistent with those used to generate previously published data [47–49], with exceptions noted in the electronic supplementary material. Whole genomic DNA was extracted using Qiagen FastStool mini extraction kits, and the 16S SSU rDNA region was targeted using custom-designed Illumina primers for salmon and groundfish, as described in [47]. Cleaned amplicons were indexed using either Illumina Nextera combinatorial indexes or Unique Dual Indexes (Illumina, Inc.), depending on when they were sequenced. Samples were sequenced on four sequencing runs (1 each in 2018, 2019; 2 in 2021) using an Illumina MiSeq next-generation sequencer (Illumina, Inc.) at Northwest Fisheries Science Center, NOAA Fisheries, Seattle, WA.

### 2.3. Metabarcoding mock communities

Two mock communities, representative of the most common killer whale prey species, were generated from genomic DNA extracted from individual vouchered fish fin or muscle samples. Whole genomic DNA was normalized to a concentration of 0.5 ng μl^−1^, based on a quantitative polymerase chain reaction (qPCR) SYBR assay of a fragment of the 16S SSU rRNA gene, before being combined in pre-determined proportions in both a general control (control 1: 15% chinook, 40% lingcod, 5% Pacific herring and 40% Pacific halibut) and a salmonid-specific control (control 2: 20% chinook, 10% coho, 20% chum, 10% rainbow trout, 10% sockeye, 10% pink salmon, and 20% Atlantic salmon). Both mock communities were sequenced with the field-collected samples in five technical replicates (totaling 10 mock mixture control samples) in 2018 (*n* = 1 replicate), 2019 (*n* = 2) and 2021 (*n* = 2).

### 2.4. Sequence alignment and QAQC

All metabarcoding sequences generated from both new and previously published libraries [47,49] were combined at this step and processed using a custom pipeline based on the dada2 package [61] in the R computing environment [62], available at https://github.com/UW-WADE-lab/Diet-variability-in-SRKW-and-SRKW. A detailed description of this pipeline can be found in the electronic supplementary materials.

Because various sources of laboratory-introduced bias can affect the observed number of reads assigned to a given species, the mock mixture control samples were used to estimate and correct for the effects of errors from, for example, amplification bias and index hopping [63]. Data generated from the two mock communities sequenced with each run were used to estimate species-specific amplification bias in a hierarchical Bayesian framework, using code that originated from Shelton *et al*. [63]. The model was run using three chains of 10 000 iterations (including a 5000 iteration warm-up) and a tree depth of 12. The estimates of amplification bias (alpha; electronic supplementary material, figure S2) were then used to estimate the true proportion of each species in each sample, and read counts were corrected according to the posterior estimate of the proportion of each species in each sample. Our mock mixtures contained 10 total species (listed above), representing the major species found in the killer whale diet as well as additional species expected in the diet (Pacific herring and Atlantic salmon). Additional species in the diet mixtures were not corrected for read count abundance but made up a small proportion of most samples and are not considered likely to contribute significantly to amplification bias [63]. Electronic supplementary material, figure S1*a*,*b*, shows estimates of amplification bias for each of the 10 species in the mock mixtures, as well as proportional diets for species included in mocks before correction, for species included in mocks after they were corrected and for all species after correction.

Once amplification bias corrections were applied to all samples, prey species were only included in downstream analyses if they represented >1% of the reads in 4 or more samples in the dataset in order to avoid potential bias from genotyping error or secondary prey (i.e. species eaten by killer whale prey). Species that occur at >1% of the diet in 1–3 samples are considered ‘minor prey species’ and are described in the results but are not included in downstream analyses because it is unknown whether these represent opportunistic prey or secondary prey.

### 2.5. Assignment of individual killer whale identity to faecal samples

Using the whole genomic DNA extracted for prey metabarcoding, we genotyped each faecal sample at 68 SNP loci on a Fluidigm platform to determine individual identity associated with each diet sample following the protocol previously described in Ford *et al*. [64]. Using only the subset of samples that were successfully genotyped at 85% or more of the 68 loci, we generated clusters based on genotype similarity using hierarchical clustering implemented in R [62]. We then compared the resulting genotype clusters to reference genotypes for all known SRKW and SARKW individuals and assigned each cluster an individual identity according to the following decision tree:

— If a single individual is known and is the sample with the greatest proportion of genotyped loci, this sample becomes the cluster representative.
— If multiple individuals are known, the known individual with the greatest proportion of genotyped loci becomes the cluster representative.
— If multiple known individuals have the same proportion of genotyped loci, the first one becomes the cluster representative.
— If there are no known individuals, the individual with the greatest proportion of genotyped loci becomes the cluster representative.
— If there are no known individuals and multiple individuals have the same proportion of genotyped loci, the first one becomes the cluster representative.

### 2.6. Data analysis

We conducted all downstream data analyses in R [62] using the phyloseq [65] and vegan [66] packages and generated graphs using ggplot [67]. After a preliminary comparison of sample composition in both biological and technical replicates, duplicate samples were removed and only the sample with the highest total read count was used in downstream analyses. Small sample sizes within each population prevented us from fitting a quantitative model to determine the effect of year, season or pod membership on diet variability within each population. Therefore, we used PERMANOVA analyses to test for statistical significance in diet differences among strata, including between populations broadly, as well as between populations in summer months. Because samples have not been collected from the SARKW during non-summer months, between-population differences in other seasonal strata could not be tested directly.

We visualized seasonal shifts in diet composition within each population by combining all samples from a given month across all years in the study and estimating the proportion of each prey species in the diet of each population, using a loess smoother with a span of 0.85 in order to reduce sensitivity to within-month variability in diet in favour of broad-scale trends. Similarly, within the SRKW population, we subdivided samples by pod and aggregated across months for all years in the study and estimated seasonal proportional abundance of each prey species, using a loess smoother with a span of 0.85.

## 3. Results

After quality filtering, a total of 185 samples were included in the final dataset (electronic supplementary material, table S1). Faecal samples were collected from the SRKW population in all months except May and July during the years 2011 and 2014–2021; samples were collected from the SARKW in May through September of 2016–2021 (electronic supplementary material, figure S1). Sample collection was opportunistic in both populations; not all months were covered in all years. Most SRKW samples were collected in September of each year (*n* = 43). SRKW samples (*n* = 98) include samples collected from 2011 to 2019 that have been included in previous publications (*n* = 79) [47–49], and 19 new samples that were collected between 2019 and 2021, in September (*n* = 7), October (*n* = 4), November (*n* = 6), and December (*n* = 2). Faecal samples collected from the SARKW population (*n* = 87) represent May (*n* = 36), June (*n* = 40), July (*n* = 3), August (*n* = 1), and September (*n* = 7). SARKW samples from this publication are also included in Olsen *et al*. [31]. Table 1 shows the average number of reads per sample at various steps in the quality control pipeline, as well as the average final number of reads per sample.

**Table 1.** Mean, minimum and maximum number of reads per sample at various points along the dada2 QAQC pipeline, used for quality filtering of all samples sequenced for [47,49], and the present study. Read counts include all samples included in the pipeline before downstream removal of samples as described in §2.

	input	filtered	denoized	merged	chimeras removed
mean	2 46 074	1 70 689	1 70 453	1 66 809	1 47 640
minimum	58	55	54	54	54
maximum	3 870 352	5 70 192	5 69 984	5 54 447	5 40 479

### 3.1. Experimental controls and technical replicates

**Figure 1.**
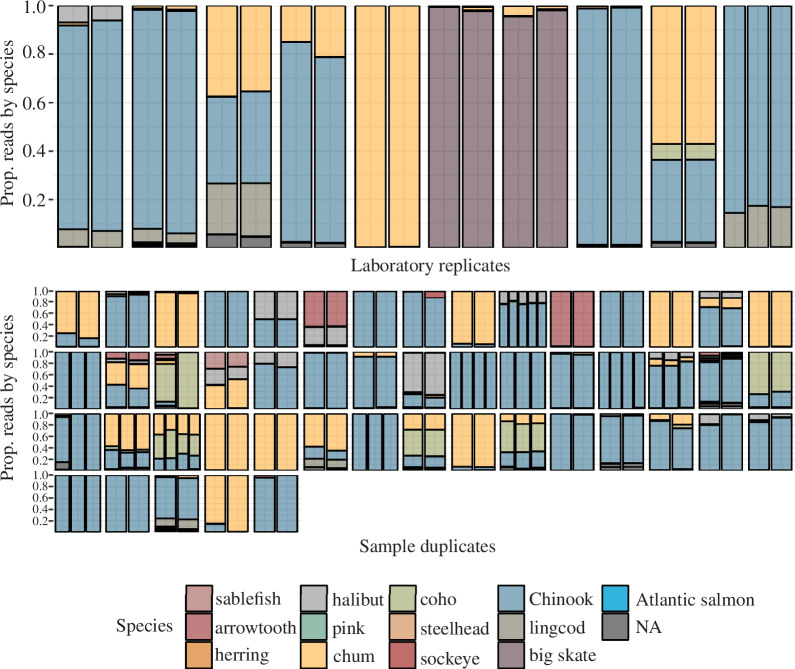
Estimated proportional abundance of common prey species across technical (top) or biological (bottom) replicates. Each bar represents an individual sample, with replicates grouped together; *x*-axis (sample name) oppressed for clarity. Top: Estimated proportional abundance of common prey species in all technical laboratory replicates included in the study, after correction for amplification bias. Bottom: Estimated proportional abundance of common prey species across biological replicates, i.e. individual whales sampled two or more times in the same day, after correction for amplification bias.

Using two mock mixtures of known species’ proportions, we found evidence of minimal laboratory-introduced bias due to, for example, differing amplification inefficiencies, index hopping, etc. (electronic supplementary material, figure S2). The estimated species-level effects of this bias, shown in electronic supplementary material, figure S2, were used to calibrate the same species in the faecal sample dataset. While amplification efficiency varied across species as expected, the effective bias contributed by variable amplification efficiencies was minor for the dominant prey species detected in killer whale faeces and did not appreciably affect the relative composition of faecal samples included in the study (electronic supplementary material, figure S3). Correcting for species-specific amplification bias did not change any of the dominant prey species identified in previous studies or preliminary analyses; therefore, these results are considered consistent with previously published studies of SRKW diet prior to the development of this new quantitative approach. Finally, our comparison of both technical and biological replicates indicates a high level of consistency in proportional abundance across all prey species with >1% abundance in each sample (figure 1).

### 3.2. Summary of prey species composition

**Figure 2.**
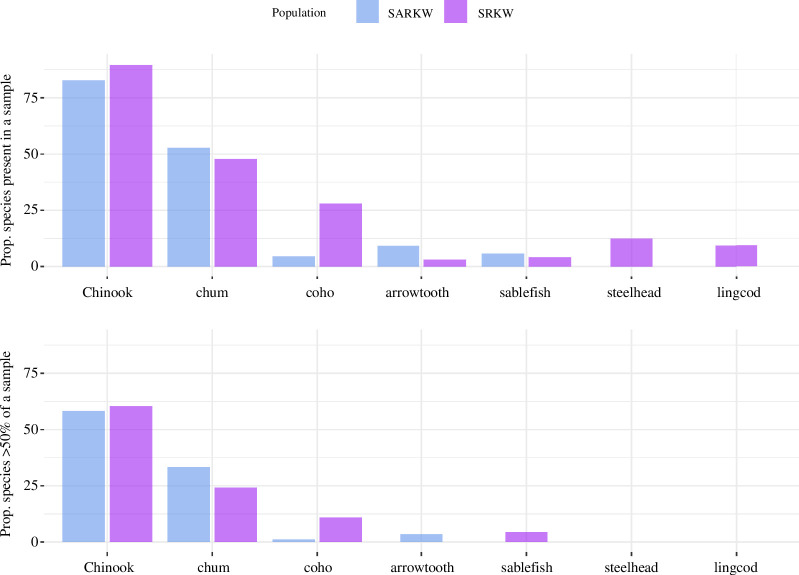
For each population, the proportion of samples in which major prey species, i.e. species that represented >1% of the sample in 4 or more samples, were present (top) or made up >50% of the sample (bottom).

Chinook salmon was the most commonly detected species in the dataset. Chinook was detected in 72 SARKW samples and 87 SRKW samples in all months in which samples were collected from both populations (figure 2). In SRKW samples in which Chinook salmon were present, they comprised an average of 64.3% (range 1.1–100%) of the prey proportion in a sample. In SARKW samples in which Chinook salmon were present, they made up an average of 69.7% (range 1.3–100%) of the prey proportion in a sample.

Major non-Chinook prey items, defined as species comprising >1% of the prey proportion in four or more samples in the dataset, varied by population and included chum salmon, coho salmon, and steelhead salmon (*Oncorhynchus mykiss*), as well as Pacific halibut, arrowtooth flounder (*Atheresthes stomias*), lingcod, and sablefish (*Anoplopoma fimbria*; figure 2). Arrowtooth flounder, identified in both populations, has not previously been identified in SRKW diet studies. In both killer whale populations, chum salmon ranked second in the number of samples in which it was present (*n*_SARKW_ = 46 [53%]; *n*_SRKW_ = 46 [47%]). Coho salmon were present in substantially more SRKW samples than SARKW samples (*n*_SARKW_ = 4 [5%]; *n*_SRKW_ = 27 [28%]). Chinook, chum, and coho salmon all make up >50% of the prey sequence reads in at least one sample collected in each population. Several of the ‘major prey species’ were present only in SRKW samples, including steelhead and lingcod; however, neither of these species were found in proportions >50% in samples from either population. Arrowtooth flounder and sablefish were present in both SARKW and SRKW samples but were detected in a larger number of samples from the SARKW population. Arrowtooth flounder were only found in proportions >50% in samples from the SARKW population, while sablefish were only found in proportions >50% in samples from the SRKW population.

Minor prey species, defined as species making up >1% of 1–3 samples, comprised four species (table 2). None of these species were detected in samples from both populations; three were present only in SRKW samples (sockeye salmon, *Oncorhynchus nerka*; big skate, *Raja binoculata*; Pacific sandab, *Citharichthys sordidus*), primarily from samples collected during winter months. One species (prowfish, *Zaprora silenus*) was detected only in faecal samples from the SARKW population, collected during the ‘early’ summer season [31]. Most of these species made up a relatively small proportion of each sample (<10%), except big skate, which had a mean proportion of 65.6% in three SRKW samples collected in January and February (*n* = 21 samples, 14%).

**Table 2.** Species making up greater than 1% of 1–3 samples in the final dataset, defined as minor prey species in this study, which are likely opportunistic or secondary prey items.

species	no. of samples	mean proportion (%)	population	month(s)
Pacific sanddab	1	1.1	SRKW	1
sockeye salmon	2	1.6	SRKW	2, 9
big skate	3	65.6	SRKW	2, 1
prowfish	3	2.1	SARKW	6

Multivariate analysis of variance in diet composition, including only major prey species, indicated small but significant variance in diet between populations (PERMANOVA *R* ^2^ = 0.016, *p*‐value = 0.017) and a greater effect among months (*R* ^2^ = 0.26, *p*‐value = 0.001) and years (*R* ^2^ = 0.14, *p*‐value = 0.001).

### 3.3. Seasonal and geographic shifts in proportional diet composition

**Figure 3.**
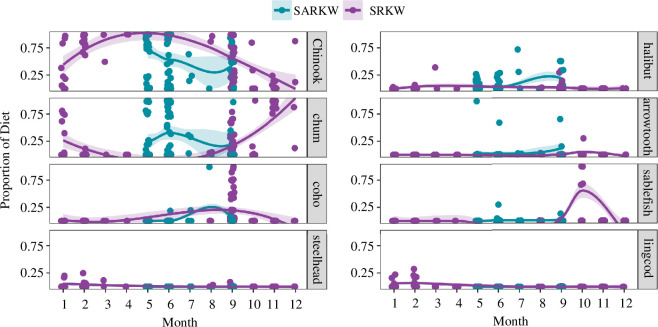
Seasonal variability in proportional abundance of major prey species in each population. Samples are aggregated by month and population across all years in the study.

**Figure 4.**
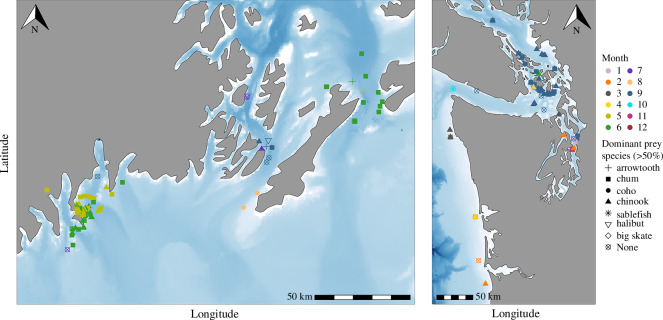
Geographic variability in the dominant prey species (>50% of sample) documented in each faecal sample included in this study. Colours represent the month each sample was collected; shapes represent the dominant prey species in that sample. Left: Geographic variability in dominant species found in samples collected from the SARKW population. Right: Geographic variability in dominant species found in samples collected from the SRKW population. Note that gut transit time is unknown, therefore samples may not have been collected in the same area where the prey were originally eaten.

In both killer whale populations, Chinook salmon dominated the composition of faecal samples collected during a portion of the summer months. Populations differed in both the timing of switching from Chinook salmon to other prey items in faecal samples, as well as which prey items replaced Chinook salmon in faecal samples (figure 3). Similarly, mapping sample collection locations according to the dominant species in each sample indicates distinct patterns of geographic variability in target prey species for each population, as well as spatiotemporal variability in sampling effort (figure 4); however, it is important to note that gut transit time is unknown in killer whales or cetaceans generally, therefore the location where samples were collected may be distant from the location where prey were consumed depending on the individual whale’s travel speed and behaviour.

In Alaska, Chinook make up the largest percentage of sample composition in May, decreasing throughout the summer months. Chum salmon were a significant proportion of sample composition throughout all months in the study period (May–September), with a slight increase in June. Chum salmon comprise a greater proportion of the diet in SARKW than in SRKW diets during the same months. Coho salmon make up a much smaller portion of faecal sample composition through most of the summer study period, increasing to approximately 25% of sample composition in August. In September, all three salmon species common in faecal samples were found in lower proportions than the rest of the summer months, and arrowtooth flounder increased in proportion. Chinook and chum dominate (>50% of species composition) samples collected in the Kenai Fjord region (westernmost SARKW sampling region) primarily during May and June (figure 4), while in Prince William Sound (eastern SARKW sampling region) samples collected during June–September are dominated by chum and a variety of salmon and non-salmonid species or have no dominant species in the sample, highlighting the spatial and temporal variability in prey preference in this population.

SRKW faecal sample prey species were dominated by Chinook salmon from March through August; this proportion began to decrease in September. Coho salmon made up an average of 25% of sample composition in September, and in the following months (October through December), chum salmon became the dominant prey item in faecal samples. Consistent with previous studies [47,49], samples collected during autumn and winter months also had a greater variety of prey species, including sablefish, lingcod, big skate, and steelhead salmon. Flatfishes such as Pacific halibut and arrowtooth flounder were not found in large proportions in SRKW faecal samples. While Chinook was found to dominate (>50% of species composition) samples collected throughout the range of SRKW, Chinook-dominated samples are most common around the San Juan Islands in late summer (August–September); samples collected in other areas and months have a greater variety of dominant species, or no dominant species (figure 4).

### 3.4. Pod-specific differences in southern resident killer whale diet

**Figure 5.**
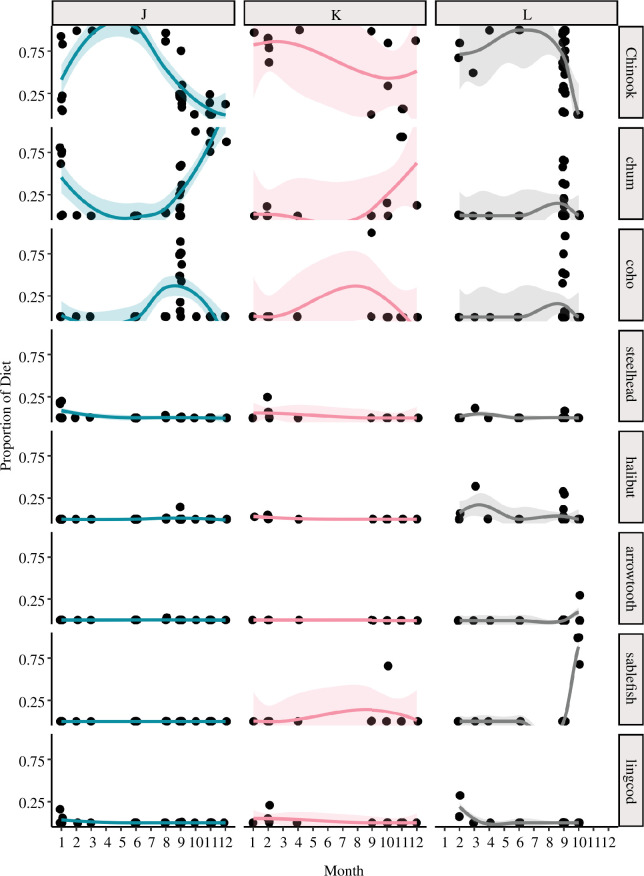
Seasonal variability in proportional abundance of major prey species in each pod within the SRKW population. Samples are aggregated by month and pod across all years in the study.

Pod data were available for a subset of 82 of 98 SRKW samples, including 36 samples from J pod, 10 samples from K pod, and 31 samples from L pod. This sample size precluded a quantitative analysis of variability among pods due to seasonal and annual diet variability within each pod; therefore, we qualitatively describe pod-level variability in diet trends by visualizing seasonal diet within each pod (figure 5). Faecal samples collected from J pod were dominated by Chinook salmon in early summer months, but Chinook began to decrease in proportion in July and was replaced by coho and then chum salmon between August and January, when Chinook began to increase in proportion once again. Steelhead contributed a small proportion to the prey items found in samples from J pod in January, and lingcod was also found in a small number of samples collected in January.

Very few samples were collected from K pod, so that in most months the diet of this pod cannot be fully characterized using faecal samples. The few samples that were collected were dominated by Chinook salmon early in the year. Coho was more prominent in the September diet of K pod compared to J pod, and both pods focused on chum in October and November. Similar to J pod, small amounts of steelhead and lingcod were found in the diet of K pod during January and February.

Samples collected from L pod were dominated by Chinook salmon through September, and this pod was the last of the three to switch to other prey types. Chum and coho make up a smaller proportion of the diet of L pod in September and October. Limited sampling in October indicates that sablefish were also present in high proportion. L pod was not sampled between the months of October and January, but a single sample from February indicates that this pod also eats lingcod during the winter months.

## 4. Discussion

This direct comparison of diet composition between two resident killer whale populations in the northeast Pacific Ocean indicates similar diet preferences at the broad scale, with population-level differences in spatiotemporal foraging strategy and resource use patterns. Temporal shifts among preferred prey species and spatiotemporal shifts in foraging location highlight the importance of various salmonid species, in addition to key fish species consumed when salmon are not targeted. At the broad scale, population-level differences in diet composition were small (PERMANOVA *R* ^2^ = 1.65), with heavy reliance on Chinook and chum in both populations, and coho to a lesser extent. Samples collected to date indicate that diet variability between the two populations is likely driven by intra-population spatiotemporal variability in foraging ecology, and both pod- and population-level variability in the timing of shifts from one target prey species to another. It is important to note that the patterns described here may be biased by opportunistic sample collection—e.g. favouring nearshore sampling and summer sampling—and overall small sample size due to the logistical difficulty of collecting faecal samples from wild populations. However, while seasonal changes in habitat use drive the spatiotemporal availability of killer whales for sampling opportunities and may result in imperfect sampling coverage in all months and regions, these patterns of habitat use are ultimately likely driven by prey availability and, as such, the patterns of prey preferences described here are likely to reflect the preferred prey targeted by killer whales in space and time.

Because variability in resident killer whale foraging behaviour varies along at least three facets (spatial, seasonal, and social), an accurate understanding of resident killer whale foraging ecology, or human-driven shifts in that ecology, will require long-term comprehensive sampling of all seasons, foraging locations, and pods within each population. Lack of representative sampling in any one of these facets may bias our understanding of resident killer whale foraging ecology and limit our ability to properly manage anthropogenic threats to these populations, which can be mitigated with an experimental sampling design that ensures representation of all pods within each population during all months of the year.

### 4.1. Comparing diet between populations

Diet preferences were broadly similar between SRKW and SARKW populations during the summer months when faecal samples were collected from both populations. During these months, Chinook make up a larger proportion of the SRKW diet overall than the SARKW diet. Chum and coho salmon make up a large part of the remainder of the diets of both populations. Flatfishes, including Pacific halibut and arrowtooth flounder, are also a small but nontrivial component of the summer diet of SARKW.

SRKW and SARKW exhibited differences in the timing of shifts among major prey items, as well as the degree to which each population relied upon various non-salmonid species as prey. Within the SARKW, the timing of shifts between species correlated strongly with the seasonally consistent use of specific foraging grounds by specific pods. Within the SRKW, all pods exhibited a tendency to converge and forage around the San Juan Islands during the late summer months in the years represented in this study [47,48]. The limited available samples from outside those months indicate a likely pod-level differentiation in foraging behaviour during autumn and winter months, resulting in diet variability across pods and the consumption of a variety of species not detected in SARKW samples. However, additional sampling from the SARKW population outside of the summer months may reveal that this population also consumes those species.

Notably, patterns of habitat use indicated by this sample set differ between the SRKW and SARKW. While samples were collected from all SRKW pods in all parts of their home range, the samples collected from the SARKW populations reveal minimal overlap in the use of two geographically distinct ranges by specific pods [31,49,68]. This difference in foraging behaviour warrants continued sampling, with a focus on increasing sampling depth during non-summer months, to fully characterize variability in the diet and foraging ecology of both populations throughout all seasons. Further, it highlights the need to estimate gut transit time in killer whales, which will facilitate a better understanding of where prey have been eaten rather than relying solely on where samples were collected. This increased resolution will be especially important to describing foraging ecology in SRKW, which travel readily throughout the Salish Sea and surrounding waters and may deposit faecal samples far from where prey were originally eaten.

Of the minor prey species detected in this study, big skate and sockeye salmon likely represent opportunistic prey items that were preyed upon by SRKW targeting other preferred prey items. These prey items were largely detected in samples collected during non-summer months in the SRKW population (table 2), suggesting that opportunistic foraging may increase in frequency outside of the summer months. Pacific sandab, observed in samples collected from SRKW, and prowfish, observed in samples collected from SARKW, may both represent secondary prey items, i.e. fishes that were eaten by fishes that then became prey to killer whales.

### 4.2. Resource tracking in a changing environment

From a resource tracking framework [69], the diet and foraging patterns of a population will reflect seasonal and temporal variability in relative abundance and availability of prey species. Consequently, foraging behaviour is affected by resource variability on six axes: abundance, timing, ephemerality, and predictability of resource patches, as well as the spatial configuration and variance of the landscape of resource patches [69]. In addition to the constraints of resource availability, foraging behaviour is affected by biological characteristics of the population, including cognitive capacity, physiology, niche breadth, trophic position, life history strategy, and social behaviour [69].

Resource tracking has been documented in a number of migratory or highly mobile terrestrial and marine species that rely on ephemeral resources [70–75]; these behaviours are often socially learned [76] and likely to integrate over years or decades of environmental variability [70]. Specialist predators are more likely to exhibit resource tracking behaviours [77], as are species with large perceptual ranges [78,79], memory capacity [7,70,78], or high capacity for social learning [80–85]. Resident killer whales exhibit all of these characteristics. The seasonal and temporal shifts in resident killer whale diet described in this paper may be the result of learned resource tracking behaviours: knowledge of the location and timing of high-quality foraging areas may be transferred vertically along matrilines over generations or horizontally within pod membership [86]. This type of social knowledge transfer provides social species with extensive generational knowledge but can slow the ability of pods to respond to rapid environmental change [11].

Within this framework, RKW foraging ecology will be affected by changes in the abundance, distribution, or life history of their prey resources. Recent climate change affects the timing and predictability of salmon runs relied upon by RKW [87–89], and large-scale marine climate events are increasing their ephemerality [59]. The net result is that RKW must contend not only with decreased availability of their primary prey resource, but also with a less predictable and more ephemeral resource landscape. The ability of RKW to respond to these changes is uncertain and could affect the survival likelihood of the population [11].

In addition to the relative availability of specific prey resources, diet preferences in both resident killer whale populations may also be related to regional differences in energy content of prey populations. In general, mature Chinook from healthy stocks have the highest fat and energy content and are the favoured prey when they are available (e.g. [90]). The observed shift in the SRKW population away from Chinook in the autumn and winter months, despite the fact that Chinook are available year-round, may be related to the relative unavailability of mature Chinook from high-energy populations in the autumn and winter [18,48,90].

During the months when large, high-energy Chinook are less readily available, RKW appear to shift their foraging strategies to focus on chum and coho. Coho are preyed upon for a only a short period of time by both populations, despite having higher energy content than chum [90]. One reason for this may be indirect competition with local pinniped populations (Steller sea lions (*Eumatopias jubatus*) and California sea lions (*Zalophus californianus*)), as well as commercial fisheries, which together have a significant impact on annual coho abundance [91]. In both regions, Steller sea lion and California sea lion populations have increased steadily since the early 2000s; in Washington, this increase has led to an approximate doubling in coho consumption since 2010 [91–93]. The majority of this consumption targets smaller size classes across all salmon species [91]; it is possible that increasing pinniped populations are removing coho salmon from the prey pool before they are large enough to be targeted by killer whales, which instead turn to target the larger but less energy-rich chum.

### 4.3. Pod-level foraging behaviour

Pod-specific social dynamics are likely to affect both diet and ranging patterns [94]. Within both populations, pods exhibit spatial segregation during the months where they have been observed and long-term stability in seasonal foraging locations [31,95] likely passed down among generations within pods. Conversely, variability in the availability of preferred prey items has been shown to affect aspects of social structure in predators [96–98], e.g. some predators form smaller groups, exhibit higher rates of solitary living, or have more extra-range excursions in response to low prey density [99,100].

In the SRKW population, sampling coverage throughout the year was limited for all pods and overall sample size was low for K pod, limiting our ability to infer pod-specific foraging behaviours and diet preferences. However, the available sample set allowed us to make qualitative observations about seasonal shifts in diet throughout the year. The pods exhibit timing differences in seasonal shifts from Chinook to other prey items, in addition to differences in preferred prey items during months when Chinook are not the primary prey. For example, results from the small number of samples collected from L pod during the non-summer months suggest that members of this pod consume sablefish in the late autumn (figure 5), and also had higher proportions of halibut than the other pods during non-summer months (figure 5). Both J and K pods targeted mainly Chinook and chum through the late autumn and into the winter months. These pod-level observations, although qualitative, indicate that the intrinsic links between social group, prey preference, and sampling locations warrant further attention to better understand intra-population variability in foraging behaviour, and may shed light on observed shifts in patterns of habitat use in recent years.

Although less is known about group structure in the SARKW population, a recent analysis of SARKW summer foraging behaviour indicates group-specific foraging site fidelity and diet differences [31]. Across a multi-year sampling effort, Olsen *et al*. [31] found that SARKW pods demonstrated site fidelity to either Prince William Sound or Kenai Fjord and described notable differences in the diet composition of animals feeding in each of those areas. SARKW diet was correlated with this spatial and social segregation, with the majority of chum-dominated samples collected in Prince William Sound from the AB, AE, AI, and AJ pods and almost all Chinook-dominated samples collected in Kenai Fjord from the AD8, AD16, and AK pods (figure 4) [31]. Pods sampled in Kenai Fjord had a diet dominated by Chinook and chum, with only minor contributions from other species. On the other hand, in Prince William Sound the diet was more varied, with greater contributions from chum, flatfishes, sablefish, and coho. Where each pod forages when not in these two areas is considered an important topic of future research effort for the SARKW population.

Parsons *et al*. [94] illustrated the importance of quantifying the strength and directionality of relationships between social structure, diet, and prey abundance in SRKW; this study highlights a continued need to elucidate those relationships to accurately describe foraging ecology in RKW populations. However, the links have not been further described due to the challenges associated with maintaining longitudinal studies as well as the inherent challenges in locating killer whales and collecting faecal samples in offshore, unprotected waters, especially during non-summer months. Elucidating this relationship would require comprehensive faecal sampling of all pods during all months of the year in tandem with ongoing efforts to monitor social structure within the population. These data are are key to developing a full understanding of the resources needed to support this endangered population; however, the scientific resources required to achieve this cannot be underestimated. The prevalence of sablefish, a fish previously unknown to be preyed upon by SRKW, in samples collected for the first time from coastal Washington during October illustrates the importance of collecting faecal samples from all pods, throughout the range of the population, and throughout the entire year.

### 4.4. Management considerations and future research

Climate variability could exacerbate fluctuations in the timing and availability of certain prey species [59], with downstream effects on predator populations. As highly mobile predators, RKW maintain large foraging grounds, which aides in mitigating variability in the abundance of key prey resources [71,101,102]. Recent research indicates that both SRKW and NRKW populations cover spatial areas large enough to maintain access to sufficient fish abundance in most years, but suggests that SRKW have a marginally higher likelihood of experiencing streaks of low abundance in Chinook prey resources than NRKW [59,101]. Climate variability and the increase in occurrence of extreme climate events disrupts the ecological dynamics of metapopulations, causing large-scale spatial synchrony across disparate fish stocks including Chinook salmon [59], which can lead to greater fluctuations in abundance and availability of this resource to its users [103–105]. While RKW are currently considered to be minimally affected by this phenomenon, contemporary field observations indicate that SRKW are shifting their seasonal occurrence within their range and shifting their diet to more available prey items (M.B.H., unpublished data). Continued climate variability could drive these populations to continue to shift their seasonal occurence, increase their range to the north or south in search of supplemental food resources, or decline under conditions of limited food availability.

In both populations, the potential for pod- or region-specific differences in diet composition illustrated in this study indicates that management strategies may need to address dietary needs on a regional or pod-specific basis throughout the year and emphasizes the importance of representing the whole of the population and its habitat when evaluating resource needs. Within each population, pod-specific diet preferences are likely to have specific effects on individual health, including body condition [37] and fecundity [106,107]. Recent studies indicate pod-specific differences in body condition fluctuations annually, which may be related to pod-specific differences in foraging behaviour and foraging success [37]; across all pods, poor body condition was linked with higher mortality. Further, intra-population variability in prey preference and foraging location (e.g. [95,108–111]) are likely to have pod-specific effects on exposure to or ingestion of toxic contaminants [112,113] as well as rates of interaction with vessels and anthropogenic noise that may alter foraging patterns or reduce foraging success [34,35].

While malnutrition and starvation can be caused by a lack of prey resources, there are alternative pathways leading to the same outcome, including disease and parasite infection. A recent pilot study of faecal parasites indicates that RKW throughout the northeast Pacific Ocean are heavily infected (>90% of samples) by Anisakid parasites, which they contract from their salmonid prey and can cause severe nutritional stress or starvation in marine mammals [114]. These parasites have a life cycle that passes through phases in both salmonids and marine mammals and have increased in salmon in recent decades [115], as well as marine mammals broadly [116]. Integrative monitoring of diet, parasite load, body condition, survival, and reproduction would provide key insight into the linked effects of diet and parasite load on survival and reproduction in resident killer whales.

## 5. Conclusions

In this study, we build on diet analyses of both SRKW [47–49] and SARKW [31] to provide a first comparison of spatiotemporal diet trends between the two populations. We further conduct a qualitative description of pod-specific diet preferences within the SRKW, which indicate the likelihood of intra-population variability in foraging behaviour and diet preference. Our results highlight the need to support comprehensive sampling of all SRKW and SARKW pods throughout the year and in all foraging locations in order to make accurate inferences about the importance of and relationships between resource availability, foraging ecology, social behaviour, and survival in these populations. As climate change, competition with other salmon consumers, and fishing pressure continue to affect the prey resources relied upon by RKW, understanding the extrinsic and intrinsic drivers of variability in diet and foraging patterns may aid in the successful conservation management of both the endangered SRKW and the SARKW populations.

## Data Availability

All raw sequence data are archived as fastq files in NCBI Genbank (BioProject ID: PRJNA1068648). Data and relevant code for this research are stored in GitHub (https://github.com/UW-WADE-lab/Diet-variability-in-SRKW-and-SARKW) and have been archived within the Zenodo repository [117]. Supplementary material is available online [118].
